# An Advanced Model to Precisely Estimate the Cell-Free Fetal DNA Concentration in Maternal Plasma

**DOI:** 10.1371/journal.pone.0161928

**Published:** 2016-09-23

**Authors:** Xiongbin Kang, Jun Xia, Yicong Wang, Huixin Xu, Haojun Jiang, Weiwei Xie, Fang Chen, Peng Zeng, Xuchao Li, Yifan Xie, Hongtai Liu, Guodong Huang, Dayang Chen, Ping Liu, Hui Jiang, Xiuqing Zhang

**Affiliations:** 1 BGI Education Center, University of Chinese Academy of Science, Shenzhen 518083, China; 2 BGI-Shenzhen, Shenzhen 518083, China; 3 Shenzhen Birth Defects Screening Project Lab, BGI-Shenzhen, Shenzhen, China; University of Warwick, UNITED KINGDOM

## Abstract

**Background:**

With the speedy development of sequencing technologies, noninvasive prenatal testing (NIPT) has been widely applied in clinical practice for testing for fetal aneuploidy. The cell-free fetal DNA (cffDNA) concentration in maternal plasma is the most critical parameter for this technology because it affects the accuracy of NIPT-based sequencing for fetal trisomies 21, 18 and 13. Several approaches have been developed to calculate the cffDNA fraction of the total cell-free DNA in the maternal plasma. However, most approaches depend on specific single nucleotide polymorphism (SNP) allele information or are restricted to male fetuses.

**Methods:**

In this study, we present an innovative method to accurately deduce the concentration of the cffDNA fraction using only maternal plasma DNA. SNPs were classified into four maternal-fetal genotype combinations and three boundaries were added to capture effective SNP loci in which the mother was homozygous and the fetus was heterozygous. The median value of the concentration of the fetal DNA fraction was estimated using the effective SNPs. A depth-bias correction was performed using simulated data and corresponding regression equations for adjustments when the depth of the sequencing data was below 100-fold or the cffDNA fraction is less than 10%.

**Results:**

Using our approach, the median of the relative bias was 0.4% in 18 maternal plasma samples with a median sequencing depth of 125-fold. There was a significant association (r = 0.935) between our estimations and the estimations inferred from the Y chromosome. Furthermore, this approach could precisely estimate a cffDNA fraction as low as 3%, using only maternal plasma DNA at the targeted region with a sequencing depth of 65-fold. We also used PCR instead of parallel sequencing to calculate the cffDNA fraction. There was a significant association (r = 98.2%) between our estimations and those inferred from the Y chromosome.

## Introduction

The discovery of cell-free fetal DNA (cffDNA) in maternal plasma has inspired various noninvasive prenatal testing (NIPT) applications [1], such as genotyping of the fetal RhD blood group [2, 3], fetal sex determination for sex-linked disorders [4], chromosomal aneuploidy detection [5–14] and monogenic disease detection [15, 16]. The fraction of cffDNA in the maternal plasma is approximately 5–20%, and this concentration is critical for the accuracy of these tests [15–19]. In some applications, whether a maternal mutation that will cause autosomal recessive diseases is passed onto the fetus can be inferred through the relative concentrations of the mutant and wild-type sequences, based on the hypothesis that the concentration of the hereditary allele will be slightly higher in the fetus [15–19]. Similarly, the cffDNA fraction is an indispensable parameter in the prenatal diagnosis of genetic disorders [20]. The cffDNA fraction in maternal plasma can also indicate the risk of pregnancy-related diseases, such as spontaneous preterm delivery [21] and preeclampsia, in asymptomatic pregnant women [20]. Thus, precisely deducing the cffDNA fraction can improve the accuracy of NIPT and may benefit the prediction of other pregnancy-related diseases.

Three types of methods have been reported to calculate the cffDNA fraction in maternal plasma. For pregnancy with a male fetus, the cffDNA fraction can be easily estimated using microfluidic digital PCR or real-time PCR targeting the Y chromosome [22, 23]. Alternatively, the cffDNA fraction can be calculated using methylated RASSF1A and unmethylated SERPINB2 sequences [22, 24]. The last approach is to employ heterozygous polymorphisms in the fetus by comparing maternal with paternal genotypes [22, 24]. However, these methods have drawbacks, such as being either restricted to pregnancies with male fetuses or requiring parental genetic information.

Recently, methods based on next-generation sequencing (NGS) have been used to calculate the cffDNA fraction by quantifying fetal-specific alleles and alleles shared between the fetus and the mother [25–27]. Prior knowledge of the maternal genotype is obtained through extra laboratory analysis. However, in the real clinical situation of non-invasive prenatal diagnosis the fetal genotype cannot be obtained beforehand and the requirement for the maternal genotype demands extra sampling and analysis efforts. Alternatively, some studies have presented an approach, using a statistical binomial model that utilizes the maximum likelihood to quantify the cffDNA fraction[18, 28]. For example, Fetal Quant directly deduces the fetal DNA concentration from targeted MPS data [29]. However, these studies did not fully prove the feasibility of inferring a cffDNA fraction lower than 8%, particularly when the sequencing depth is less than 100-fold, because it was difficult to distinguish sequencing errors from the fetal chromosome when the sequencing depth or cffDNA fraction was low in maximum likelihood fitting.

In this study, we present a statistical model of a Gaussian mixture to calculate the cffDNA fraction directly from targeted MPS data of maternal plasma DNA. The different types of single nucleotide polymorphisms (SNPs) were assumed to be in a Gaussian distribution in this method. According to the confidence interval of the Gaussian distribution, three boundaries were added to capture effective SNP loci in which the fetus was heterozygous and the mother was homozygous to calculate the cffDNA fraction. Although some effective points should be divided into sequencing with a low sequencing depth or low cffDNA fraction, the bias could be fitted because the boundary between target points and sequencing errors is fixed, and the deviation is regular to larger than the standard. The method that we propose could accurately estimate cffDNA fraction when it was more than 3% without using any genotype information from the fetus or parents. To decrease the sequencing costs even further, we successfully reduced the depth of sequencing to 65-fold without affecting the reliability of the results of the cffDNA fraction. Furthermore, we replaced parallel sequencing with PCR to decrease costs and showed that this method also precisely calculated the cffDNA fraction. There was a significant association (r = 98.2%) between our estimations and those inferred from the Y chromosome.

## Materials and Methods

### Overall design

**Fig 1:**
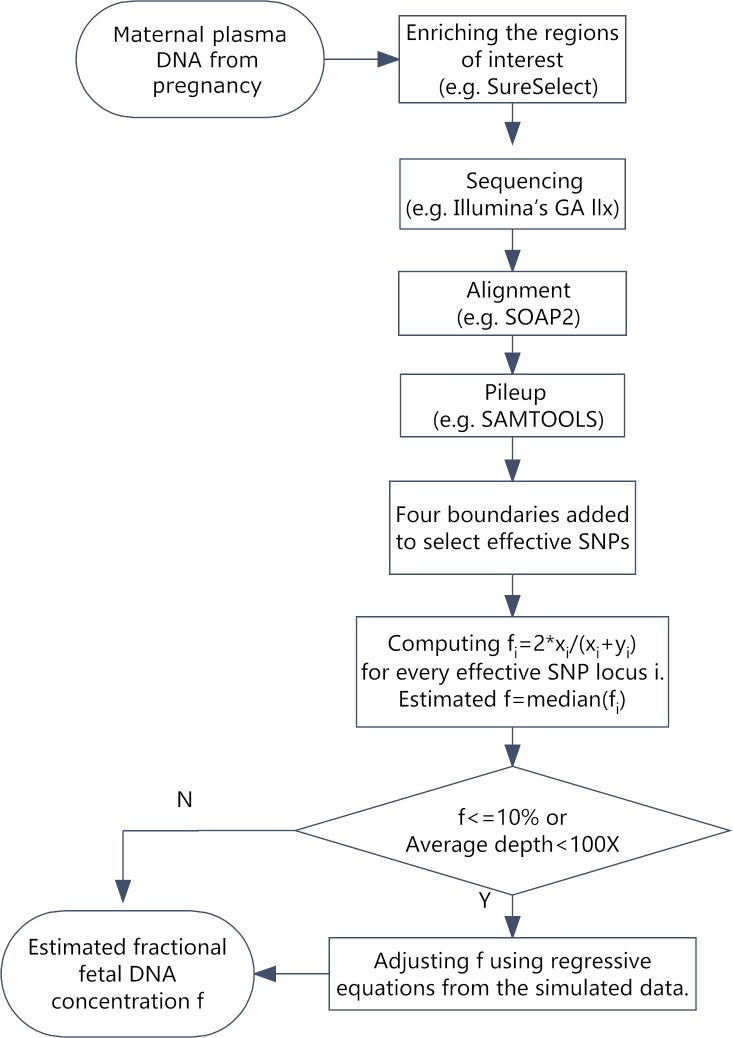
The workflow to estimate the fractional fetal DNA concentration. The first step is to extract free DNA from plasma samples from pregnant women. Then, the regions of interest in the extracted DNA fragments are enriched using a crossing system. The next step is to prepare the DNA sequencing library and sequence the samples. After sequencing, these reads are matched to a reference genome (Hg19) using SOAP2. The pileup files of the matching are generated by SAMTOOLS for the covered regions. Each point of the targeted zones is calculated following Formula 1, and the median value is considered the cell-free fetal DNA concentration. If the depth is more than 100-fold and the cff-DNA is larger than 10%, we can obtain the cff-DNA concentration directly. Otherwise, we can fit the result using an equation.

Effective SNPs that were homozygous in the maternal genome and heterozygous in the fetal genome were applied to estimate the cffDNA fraction in the maternal plasma. To capture all potential effective SNPs, the SNPs were classified into four categories of maternal-fetal genotype combinations [29]; three boundaries were added to separate the categories and make the selection. Among the effective SNPs, each locus was used to propose a corresponding value of the cffDNA fraction; the median value was considered the final cffDNA fraction. We performed a depth-bias correction, using simulated data and the corresponding regression equations for adjustments because a low sequencing depth (<100-fold) or low cffDNA fraction (<10%) led to insufficient fetal-origin alleles in the effective SNPs, which was the key factor of our method. The flowchart of our proposed method is presented in Fig 1.

### Multiplex PCR target enrichment system

To enrich the regions of interest from the whole genome in this study, we introduced a method based on anchored multiplex PCR to capture target regions in addition to the array-based hybridization enrichment system. The study protocol was approved by Institutional review board of BGI-Shenzhen.

The clinical NIFTY (Non-invasive Fetal Trisomy Test) libraries for the sequencing platform BGISEQ-1000 from BGI-Diagnostics (Shenzhen, Guangdong, China) were applied. The biotin marker was introduced during the library construction procedure, and streptavidin beads were used to separate the single-stranded DNA (ssDNA) as the amplification template. The primer pool was added to the ssDNA to start the first round of the PCR (linear amplification). Then, the DNA was denatured at 95°C to elute the single-stranded amplification products. The primer pool and universal adapter primer were added to the products, again using the first round amplification products as the templates to start the second round of the PCR (exponential amplification). The final double-stranded DNA products were the enriched sequences of the target region.

### SNP selection

According to the maternal-fetal genotype combinations, the SNPs were classified into four types. These types can be described as AAaa, AAab, ABaa and ABab, where the upper-case and lower-case letters represent the maternal and fetal genotypes, respectively [29]. In Type 1 (AAaa) the mother and fetus were both homozygous for the same allele; in Type 2 (AAab) the mother was homozygous but the fetus was heterozygous; in Type 3 (ABaa) the mother was heterozygous but the fetus was homozygous and in Type 4 (ABab) both the fetus and the mother were heterozygous. In the study, the cffDNA fraction was calculated from category AAab using Formula 1. The depth of sequencing in every effective SNP locus can be calculated by adding the counts of the major and minor alleles (denoted x and y, respectively); then, the median value is considered the sequencing depth of the sample (denoted D).

**Fig 2:**
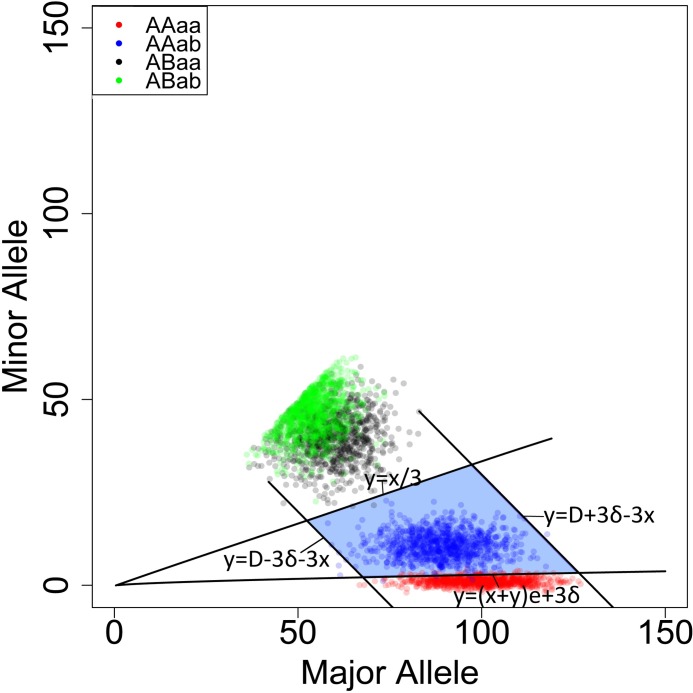
The distribution of SNPs in maternal plasma The x-axis and y-axis show the depth of major (A/a) and minor alleles (B/b), respectively. *y* = *D* + 3*δ* − *x* and *y* = *D* − 3*δ* − *x* are simplified as the form of x+y = u+3*δ, where D denotes the depth of the SNP loci. (x+y)*e of Line 2 *y* = (*x* + *y*)*e* + 3*δ* represents the depth of minor alleles of category 1 (AAaa) caused by sequencing errors. The expectation (u) of the normal distribution is equal to the variance (δ^2^) when the Poisson distribution is approximately close to the normal distribution. Thus, δ^2^ is approximately equal to the mean of (x+y) in the equation *y* = (*x* + *y*)*e* + 3*δ*. Similarly, δ^2^ will be roughly equal to D in the functions *y* = *D* + 3*δ* − *x* and *y* = *D* − 3*δ* − *x*.

As shown in Fig 2, each SNP was represented by a specific point. The x-axis and y-axis represent the counts of the major alleles (A/a) and the second most common alleles (B/b), respectively. Only the effective SNPs (the blue area) were used to estimate the fetal DNA concentration. Formula 1 was used to calculate the cff- DNA concentration (f) at each effective SNP locus.

$$f=\frac{2y}{x+y}$$ (1)

Because the points of the four categories had their own properties, four boundaries were chosen to select effective SNPs. First, by assuming that the maximum cffDNA fraction (f) achieves 50%, we obtain a boundary of *y* = *x*/3. According to previous studies [7, 11], the cffDNA fraction is unlikely to be more than 50% in the plasma of pregnant women. When f is between 0 and 50%, the expectation of the minor allele at ABaa is between x/2 and x/3. Because the expectation of the minor allele at ABab is x/2, Line 1 of *y* = *x*/3 is applied to exclude SNPs of category 3 (ABaa, the black area) and category 4 (ABab, the green area). If sequencing errors do not exist, there are no minor alleles for the SNPs of Category 1 (AAaa). In reality, many of the AAaa SNP loci cause deviations in the deduction of the cffDNA fraction due to sequencing errors. In previous studies, sequencing errors were assumed to obey a binomial distribution [30]. However, we can assume that the sequencing error is subject to the normal distribution, based on the central limit theorem and approximation theory [30]. According to the nature of the normal distribution, 99% of the observed values fall within the scope of three standard deviations from the average[31]. To eliminate the influence of sequencing and alignment errors, Line 2 of *y* = (*x* + *y*)*e* + 3*δ* is added to consider the maximum counts of the minor alleles. Moreover, the Poisson distribution is similar to the binomial distribution [32] and applies well in this capacity according to the following rule of thumb: the sample size n should be equal to or larger than 20, and the probability of a single success (p) should be smaller than or equal to 5%. If n≥00, then the approximation is excellent if np is also ≤10. In the study, we filtered out reads with quality less than Q20; therefore, the sequencing error equals 1% and we assumed e is 1%. We also assumed that the variance (δ^2^) is equal to the expectation (μ) of the depth of each SNP locus (x+y) because it is close to the Poisson distribution. Additionally, the left and right boundaries were set to three SD deviations from the sequencing depth of the sample (D) as (Line 3, *y* = *D* − 3*δ* − *x*) and (Line 4, *y* = *D* + 3*δ* − *x*), respectively. Previous studies indicated that the depth of sequencing was subject to a Poisson distribution[33, 34]. According to the statistical analysis[32], the normal approximation of the Poisson distribution is adequate when the mean of the Poisson distribution is at least 5. In this study, the mean of the minor allele at AAab is 5 when the depth of sequencing is 200-fold and f is 5% because the cff-DNA concentration of 95% of the maternal plasma samples is more than 5% [29]. With the supposition that the distribution of AAab (the most common allele or minor allele) is subject to a normal distribution and the variance (δ^2^) is equal to the expectation (D), the targeted zone (Fig 2) is established based on four boundaries and the effective SNPs are found. Finally, Formula 1 is used to calculate f of each SNP locus in the targeted zone and the median is considered as the estimated f.

### Boundary adjustments and depth-bias correction

In our method, the key factor influencing the precision of the estimated f lies in the sequenced fetal-origin alleles in the effective SNPs. Our proposed method does not work well when the cffDNA fraction or the sequencing depth is relatively low because the usable effective SNPs are not adequate. A higher sequencing depth will not help to calculate f when the cffDNA fraction is low because fetal alleles are more detectable in this situation, suggesting that our method has a depth bias. Another explanation is that the selective standard of the four boundaries is so tight that some effective SNP loci (AAab) will run out of the targeted zone and mistakenly switch to the AAaa zone. In this case, the estimated value will be higher than the real value. To eliminate this influence and optimize the model, boundary adjustments and depth-bias corrections were made. When the depth is less than 100-fold, we switch Line 4 from *y* = (*x* + *y*)*e* + 3*δ* to *y* = (*x* + *y*)*e* + 2*δ*; then, 95% of the AAaa points will fall within the confidence interval (U − 2δ,U + 2δ) [31]. To correct the depth bias, we established a computationally simulated dataset as the standard that was composed of a set of SNP loci with the allelic counts of major and minor alleles. Each SNP locus of the synthetic dataset was simulated with the following criteria:

1. The number of SNP loci used was 10,000 for all simulated samples. Following the previous study, the ratios of the maternal-fetal genotype combinations were set to 0.7, 0.1, 0.1 and 0.1 for AAaa, AAab, ABaa and ABab respectively [29]. The predefined median sequencing depth for each SNP site (denoted D) was set similar to that of the experimental maternal plasma data. In the previous study, the sequencing error of Hiseq2000 was 0.26%[33]; therefore, the expectation of AAaa is 0.0026*D.
2. The standard cffDNA fraction (f) was predefined from 0.5% to 25% with an increment of 0.5% for each simulated dataset.
3. The major and minor allelic counts at the SNP locus i (denoted xi and yi, respectively) followed the normal distribution. In the previous study, the allelic count of each locus followed the binomial distribution [29, 30, 35]. According to the De Moivre Laplace theorem, the shape of the binomial distribution begins to resemble that of the normal distribution, as the number of SNP loci grows larger. However, the sequencing depth was subject to a Poisson distribution. Therefore, in our assumptions the major and minor allelic counts of each locus were subject to the normal distribution, and the expectation (μ) of the normal distribution was equal to the variance (δ2).

Following the above criteria, we generated synthetic datasets using an R script. Using the method described previously to calculate the estimated f from the synthetic datasets, we obtained the regressive equations of the estimated f and the standard f of the synthetic datasets (S1 Table), which corrected the estimated value of the cell-free fetus DNA concentration in the plasma samples. Consequently, when the estimated f of the maternal plasma sample was obtained from the previous analysis, the adjusted value of f was corrected from the synthetic datasets at the same depth, using the equation at the same depth.

### Performance evaluation with simulated data

To establish simulated plasma data, we blend maternal and fetal reads from the maternal (200-fold) and fetal (130-fold) sequencing data generated after massive parallel sequencing and alignment to the human reference genome Hg19 using SOAP2 [36]. To calculate the counts of the major and minor alleles at each locus, we utilized SAMTOOLS to generate pileup results of the simulated plasma data.

The standard value of (f) in the simulated data was the median value of the cffDNA fraction, estimated using the SNP loci of AAab. First, we generate depth-related simulated plasma (150-fold) by blending the maternal and fetal reads to evaluate our method. The standard of the cffDNA fraction of the simulated plasma samples range from 3% to 20%.

### Application in clinical samples

The plasma sample data was collected from 18 pregnant mothers, including 5 samples that were published in previous publications [37]. The selected SNP sites were distributed on 22 normal chromosomes and their sub-high base frequencies were greater than 0.4 in the dbSNP 134 library. The designed probes covered 761,159 bp of the human reference genome (HG19, NCBI build 37). This version (called Mini-I) was used for our samples from families p-1, p-2, p-3, p-4, p-10, p-13, p-15, p-16 and p-17. We also designed an updated version called Mini-II, which covered 4,525 SNPs and 1,508,117 bp of the human reference genome (HG19, NCBI build 37) using families p-5, p-6, p-7, p-8, p-9, p-11, p-12 and p-14 [37]. After sequencing, we used SOAP2 [36] to align all sequenced reads to the human Hg19 sequence. The base pileup files were generated by SAMtools [38]; the major alleles and minor alleles were generated to estimate the cffDNA fraction, based on the mixed normal distribution model.

## Results

### Evaluation using simulation datasets

**Fig 3:**
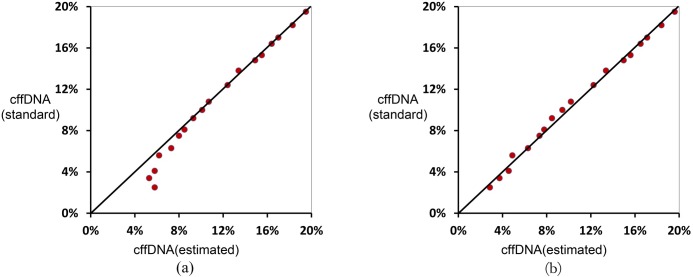
The estimated fractional fetal DNA concentration before and after the fitting correction. The x-axis and y-axis represent the estimated value and standard value of the cell-free fetal DNA concentration in the plasma, respectively. (a) Most estimated values are close to the standard values. However, the estimated value was higher than the standard value when the standard value was less than 8%. (b) The bias was corrected through the equation (S1 Table) generated by the corrected model. Finally, the deviation was reduced to a satisfactory level when most of the estimated values and standards were in the diagonal line.

The cffDNA fraction of the simulated plasma samples ranged from 3.5% to 19.5%. The results generated using the SNP loci in our targeted zone ranged from 7.6% to 19.5%. Furthermore, the results showed that a majority of the points were located around the slanting line, indicating that the estimated results were close to those of the standard values except when the cffDNA fraction was lower than 8% (Fig 3). As shown in Fig 3, the deviation became larger as the cffDNA fraction reduced, indicating that necessary adjustments should be performed to fix the bias and correct the estimated results. Using the well-optimized equation (S1 Table) generated by the corrected model, the derivation caused by the bias was decreased to a satisfactory level.

### The sequencing depth and the number of SNP loci required in the method

Next, we examined the factors that would influence the accuracy of the cffDNA fraction inference in this model. The results of our study showed that the depth of sequencing and the number of SNP loci used in the model were two important factors. First, the accuracy of the cffDNA fraction deduction was affected by the detectability of the minor alleles at each SNP locus, which was critical for the categorization of the maternal–fetal genotype combination. If the sequencing depth is not sufficiently deep, the minor alleles cannot be discovered by the sequencing reaction. Second, it was clear that the increases in the SNP quantity improved the precision of detecting the fetal DNA concentration. However, the need for more SNP loci requires more targeted genomic region sequencing and accordingly increases the sequencing cost. Consequently, it was critical to know the minimum sequencing depth requirements and the number of SNP loci required for the accurate cffDNA fraction deduction.

To investigate the required depth of SNP loci for accurate deduction of the cffDNA fraction, we implemented a simulation analysis with the cffDNA fraction fixed at 3%, which was the lowest fetal DNA concentration that the model could precisely predict, using simulated plasma data. We also used 11,000 predefined SNP loci to ensure sufficient points in the targeted zone, assuming that the allelic account followed the normal distribution. Next, we produced datasets with diverse sequencing depths (ranging from 40-fold to 200-fold at the fixed cffDNA fraction of 3.5%). We calculated the degree of deviation (denoted e_1_%) with Formula 2 to examine the relationship between the accuracy of the cffDNA fraction deduction and the sequencing depth.

$$e_{1}\%=|standard\;f-deduced\;f|$$ (2)

The result showed that e_1_% decreased with the increasing sequencing depth. We also found that e_1_% would be less than 1% when the depth of the sequencing was larger than 65-fold (S1 Fig).

$$e_{2}\%=\frac{\left|standard\;f-deduced\;f\right|}{standard\;f}$$ (3)

To investigate how the deduction accuracy was affected by the number of SNP loci, we fixed the sequencing depth at 65-fold and the cffDNA fraction at 5%, 10% and 20%, respectively. We designed the degree of deviation (e_2_%) with Formula 3 to measure the relationship between the accuracy of the cffDNA fraction deduction and the number of SNP loci. We found that the relative deviation was less than 1% when more than 30 SNP loci were in the targeted zone. Then, we calculated the minimal number of SNPs needed and found that 7857 SNPs were required when the fetal DNA concentration was 3%. As expected, the minimal number of SNPs required in our approach decreased when the cffDNA fraction increased. Fewer than 2000 SNPs were needed when the cffDNA fraction was more than 5.6% (S2 Fig). In conclusion, more SNP loci were needed during early pregnancy because the cffDNA fraction was then generally low; in contrast, the number of SNP loci was not so demanding in late pregnancy due to the rising cffDNA fraction.

### Evaluation using capture sequencing datasets

The experimental dataset was comprised of maternal plasma samples from 18 pregnancies, including 5 samples reported in previous publications [37]. We obtained a more accurate cffDNA fraction when the sequencing depth of the samples was more than 100-fold (Table 1). However, the estimated value was larger than the standard value when the depth of the samples was less than 100-fold (Table 1). Therefore, we revised the estimated value with the correction model when the depth was less than 100-fold. Prior to correction, the median deviation was 1.8% (0.6%-3.6%) and was reduced to 0.65% (0.2%-2.1%) after correction (Table 1).

**Table 1:** Estimated and corrected fractional fetal DNA concentrations in the 18 samples.

Samples	Depth (fold)	Sensitivity (%)	Specificity (%)	Standard CFF^a^ (%)	Estimated CFF^a^(%)	Corrected CFF^a^ (%)
p-1	54	43.4	80	7.7	11.3	7.9
p-2	55	82.6	86.3	23.7	22.4	21.6
p-3	58	67.6	81.9	12.9	14	12.3
p-4	60	66.7	96.6	14.6	16.3	15.3
P-5	65	32.9	67.2	9.9	11.8	9.6
P-6	73	46.1	88.3	9.6	12.3	10.7
P-7	78	64.9	86.9	13.3	13.9	12.9
P-8	79	39.7	87.8	8.80	11.3	9.6
P-9_1	114	69.7	93.8	19.6	19.6	-
P-9_2	137	58.4	77.1	18.8	17.7	-
P-10	136	84.2	91	14.2	14.6	-
P-11	142	91.2	92.6	17.6	17.5	-
P-12	145	59.3	96.2	16.5	16.5	-
P-13	152	89.4	98.1	17.1	17.1	-
P-14_1	165	48.1	94.7	11.1	11.8	-
P-14_2	205	40.5	79.3	10.4	10.1	-
P-15	203	88.7	95.5	15.7	15.6	-
p-16	290	42.9	97.3	23.1	23.2	-

-: unobtainable data; CFF^a^: fractional fetal DNA concentration.

**Fig 4:**
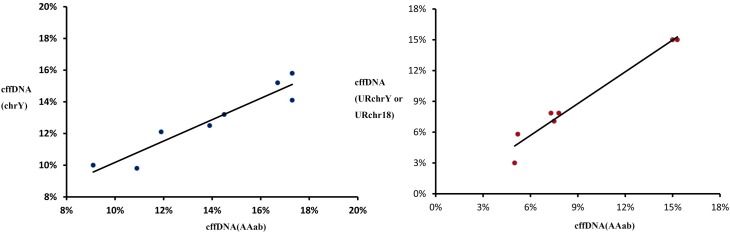
The estimated fractional fetal DNA concentration using capture sequencing data and MT-PCR data. (a) The x-axis represents the estimated fractional fetal DNA concentration, using polymorphic alleles by capture sequencing. The y-axis represents the estimated fractional fetal DNA concentration, using SNPs from the Y-chromosome with capture sequencing. (b) The x-axis represents the estimated fractional fetal DNA concentration using polymorphic alleles by MT-PCR. The y-axis represents the estimated fractional fetal DNA concentration using URchrY or URchr18 counts by MT-PCR.

To evaluate the feasibility of our approach, we compared the result of the targeted zone with the result from estimating f through the Y-chromosome. In this study, we included 8 plasma samples (p-7, p-8, p-10, p-11, p-12, p-13, p-14_1, and p-14_2) from pregnancies with male fetuses. We calculated f by the depth of the locus in the Y chromosome annotated in dbSNP 134. The median of the cffDNA fraction estimated by AAab was 13% (range 9.1–17.3%) and there was a significant association with the fraction determined by the Y chromosome counts (r = 0.935; p < 0.0001; Fig 4).

### Evaluation using the MT-PCR datasets

Finally, we amplified plasma DNA from 7 pregnant women using 909 pairs of multiplex PCR primers and then sequenced the amplified products using Ion Proton. These 7 samples included 5 plasma samples from pregnancies with male fetuses and 2 plasma samples from pregnancies with trisomy 18. The median of the cffDNA fraction estimated by AAab was 9.0% (range 3–15.0%), and there was a significant association with the fraction determined by the URchrY or URchr18 counts (r = 0.9817; p < 0.0001; Fig 4).

## Discussion

In NIPT, the cffDNA fraction is significant not only for diagnostic algorithms but also to predict the risks of spontaneous preterm delivery and preeclampsia [21]. Here, we describe a normal mixture model to deduce the cffDNA fraction, using targeted sequencing data from maternal plasma DNA.

Our proposed probabilistic approach could make precise estimations of the cffDNA fraction without previous information on the fetal or parental genotype. We used massive parallel sequencing to obtaini sequencing data from high depth sequencing more cost effective. This probabilistic model could be a valuable method to improve the accuracy of NIPT, which requires a precise determination of the cffDNA fraction.

This study assumed that the SNPs were distributed throughout the whole genome randomly, which was one limitation of this method. In reality, the SNP distributions exhibit some preferences. For example, the neighboring-nucleotide patterns of transitions dominated by the hyper-mutability effects of CpG dinucleotides and transitions were four times more frequent than transversions among the substitution mutations [39]. Another potential limitation of the model is that it assumes that sequencing errors are stochastic. However, the sequencing error was biased and happened more frequently for bases of reads with low quality and reads near the 3’ end. AC and GT miscalls during base calling were meaningfully over-represented [38]. In the model, alignment errors and the allele count bias were introduced, which were not entirely taken into account [36, 40]. However, the SNP loci (11,000 and 4500) of the chip in this experiment were scattered throughout 22 autosomes that were used to predict the fetal DNA concentration. Thus, the specific bias caused by certain SNPs may donate slightly to the current model because the median of the targeted zone is chosen.

The accuracy of the cffDNA fraction assumption will be further improved when we can obtain higher quality sequencing reads through the continual development of sequencing technologies and bioinformatics alignment software. The main problem in this study is to deduce the fitted curves of the mixture model. In the future, we can use statistical approaches (e.g., the random forest algorithm) with more maternal plasma samples to optimize the fitted curves that are crucial for this method to establish a more reliable and effective method to detect the cffDNA fraction in the maternal plasma.

## Supporting Information

S1 Fig The lowest depth required to estimate the fractional fetal DNA concentration. The degree of deviation will decrease with the increasing sequencing depth of the simulation plasma.
(TIF)

S2 Fig The lowest number of SNPs required to estimate the fractional fetal DNA concentration. The relative deviation decreased with the increasing number of SNPs in the target.
(TIF)

S1 Table Equation of fitting correction at different depth. The correction equation derived from the data with the computer simulation of the different depth, used to correct the detected fetal concentration.
(XLSX)
