# Supplementary figures and images for: An Advanced Model to Precisely Estimate the Cell-Free Fetal DNA Concentration in Maternal Plasma
Source: PLoS One. 2016 Sep 23;11(9):e0161928. doi: 10.1371/journal.pone.0161928 (PMC5035032; doi:10.1371/journal.pone.0161928)

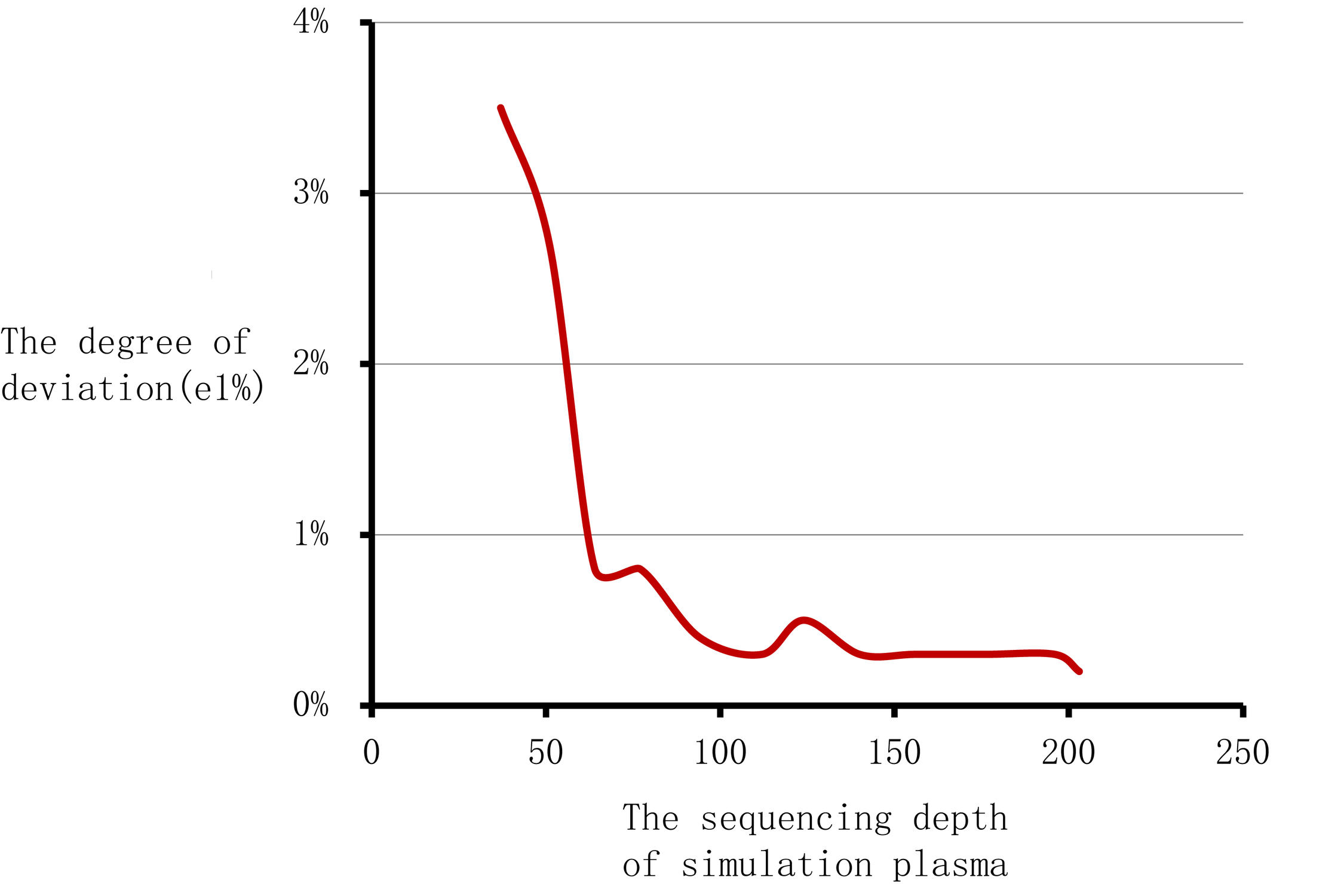

Supplement: S1 Fig — The degree of deviation will decrease with the increasing sequencing depth of the simulation plasma. (TIF) [file pone.0161928.s001.tif]

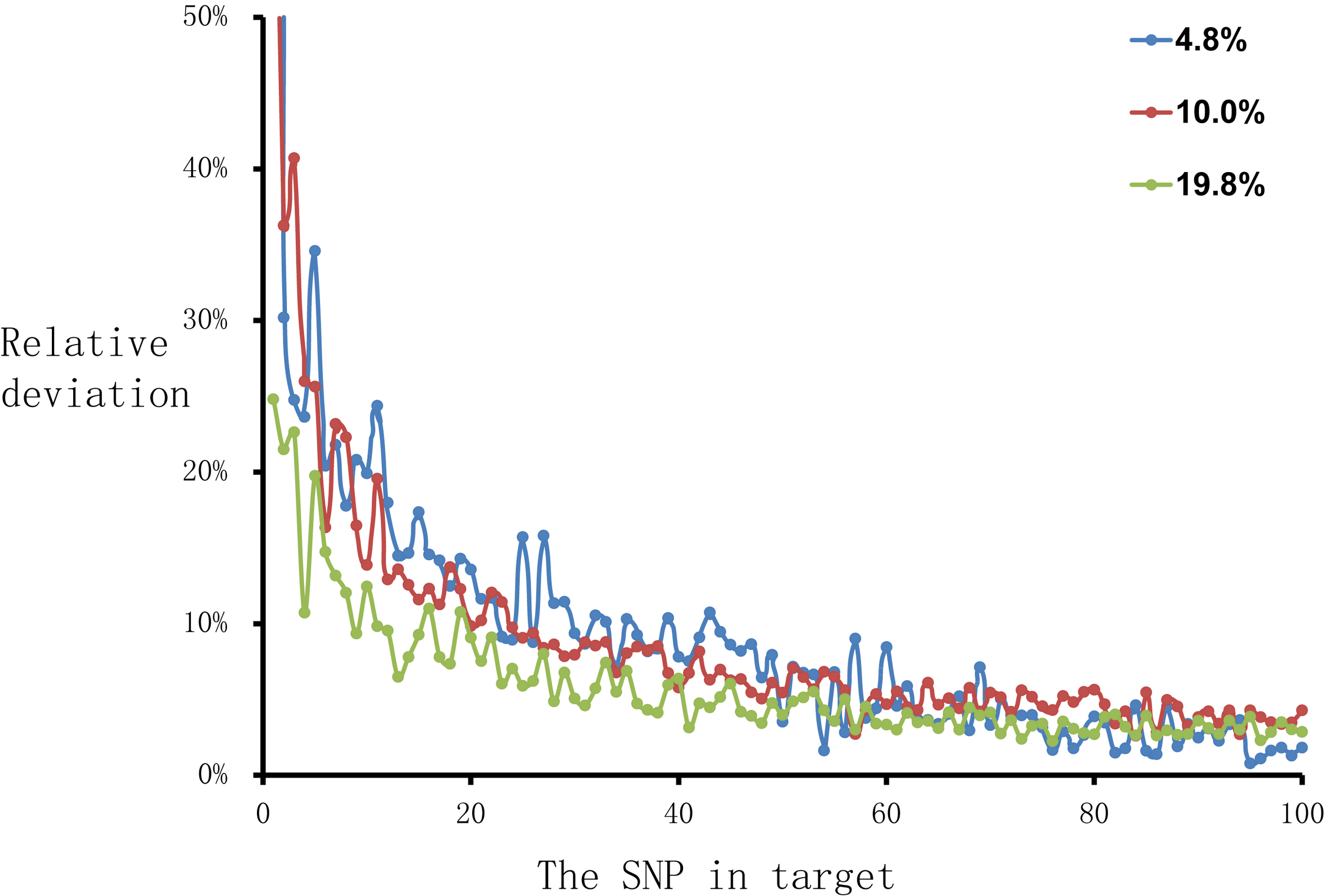

Supplement: S2 Fig — The relative deviation decreased with the increasing number of SNPs in the target. (TIF) [file pone.0161928.s002.tif]
